# Supplementary material for: Co-Exposure of Polycyclic Aromatic Hydrocarbons and Phthalates with Blood Cell-Based Inflammation in Early Pregnant Women
Source: Toxics. 2023 Sep 25;11(10):810. doi: 10.3390/toxics11100810 (PMC10611080; doi:10.3390/toxics11100810)
Supplement: Supplementary file 1 [file toxics-11-00810-s001.zip › toxics-2611628-supplementary.pdf]

Table S1 Demographic characteristics of women in and out of early pregnancy

Variables		Included group(n=318)	Total group(n=393)	*P*
age		26.22±4.7	26.38±4.7	0.66
Pre-pregnancy BMI (kg/m2)		22.10±4.22	22.06±4.24	0.894
Level of education	High school degree or below	232 (72.95%)	276 (70.2%)	0.423
	College degree or above	86 (27.04%)	117 (29.8%)	
Ethnic	Minority	6 (1.8%)	9 (2.3%)	0.71
	Ethnic Han	312 (98.11%)	384 (97.7%)	
Marital status	Married	277 (87.11%)	346 (88.0%)	0.923
	Spinsterhood	39 (12.26%)	45 (11.5%)	
	Divorced	2 (0.62%)	2 (0.5%)	
Smoking		20 (6.28%)	29 (7.4%)	0.568
Drink		27 (8.49%)	35 (8.9%)	0.845
Passive smoking		313 (98.43%)	385 (98.0%)	0.647
Activities	Little or no	167 (52.52%)	223 (56.7%)	0.468
	1-2/week	41 (12.89%)	42 (10.7%)	
	≥3/week	110 (34.59%)	128 (32.6%)	
WBC		8.09 (6.80, 9.58)	8.31 (6.90, 9.56)	0.805
lymphocyte		1.80 (1.50, 2.18)	1.88 (1.50, 2.20)	0.869
neutrophile granulocyte		5.73 (4.60, 6.94)	5.86 (4.67, 6.96)	0.769
monocyte		0.40 (0.32, 0.48)	0.42 (0.33, 0.48)	0.307
platelet		241.5 (202, 274)	244.4 (204.5, 274)	0.769
NLR		3.11 (2.43, 3.94)	3.31 (2.44, 3.93)	0.681
PLR		130.50 (109.44, 155.56)	136.9 (109.72, 155.44)	0.979
SII		727.00 (552.05, 942.74)	813.95 (554.65, 952.35)	0.882

Table S2. Retention time, ion pair, collision energy, determination coefficient, recovery rate, and detection limit of each metabolite.

Chemical	RT (min)	Precursor Ion	Product Ion	CE (eV)	ISTD	Determination coefficient (R^2)	LOD (μg /L)	Recovery (%)	RSD (%)
1-OHNap-D7	15.987	223	208	15	N/A	N/A	N/A	N/A	N/A
1-OHNap	16.043	216	201	15	1-OHNap-D7	0.9998	0.005	107.05	1.4
2-OHNap	16.547	216	201	15	1-OHNap-D7	0.9999	0.0027	105.71	2.27
9-OHFlu	20.231	254	165	20	1-OHNap-D7	0.9998	0.0023	94.15	2.85
2-OHFlu	22.471	254	239	15	1-OHNap-D7	0.9998	0.0029	91.99	3.18
4-OHPhe	25.209	266	235	25	1-OHNap-D7	0.9999	0.0167	90.08	5.01
9-OHPhe	25.211	266	73	25	1-OHNap-D7	0.9982	0.0044	105.01	4.99
1-OHPhe	25.685	266	73	25	1-OHNap-D7	0.9998	0.0071	81.09	4.96
3-OHPhe	25.854	266	73	25	1-OHNap-D7	0.9999	0.0095	84.5	5.48
2-OHPhe	26.702	266	73	25	1-OHNap-D7	0.9999	0.0115	68.19	2.41
1-OHPyr	30.857	290	73	25	1-OHPyr-D9	0.9999	0.0003	100.3	1.97
1-OHPyr-D9	31.229	299	73	25	N/A	N/A	N/A	N/A	N/A
MMP	16.731	237	89	15	MEHP-C4	0.9986	0.0375	80.45	8.37
MEP	17.837	251	75	15	MEHP-C4	0.9972	0.0288	102.31	6.15
MIBP	17.84	223	75	15	MEHP-C4	0.9988	0.0023	99.71	4.95
MBP	19.785	223	75	15	MEHP-C4	0.9982	0.0015	92.64	4.77
MOP	20.426	223	73	15	MEHP-C4	0.9997	0.0625	100	3.15
MEHP	21.669	221	73	15	MEHP-C4	0.9999	0.0326	112.59	3.59
MEOHP	24.633	221	73	15	MEHHP-C4	0.9986	1.0714	105.16	4.14
MEHP-C4	27.619	225	73	15	N/A	N/A	N/A	N/A	N/A

MEHHP-C4	28.154	225	73	15	N/A	N/A	N/A	N/A	N/A
MEHHP	29.367	221	73	15	MEHHP-C4	0.9998	0.0103	104.21	3.67
MECPP	31.667	221	73	15	MEHHP-C4	0.9847	23.4375	134.28	54.4

Abbreviations: RT, retention time; CE, collision energy; ISTD, internal standard substance; LOD, limit of detection; RSD, relative standard deviation; N/A, not

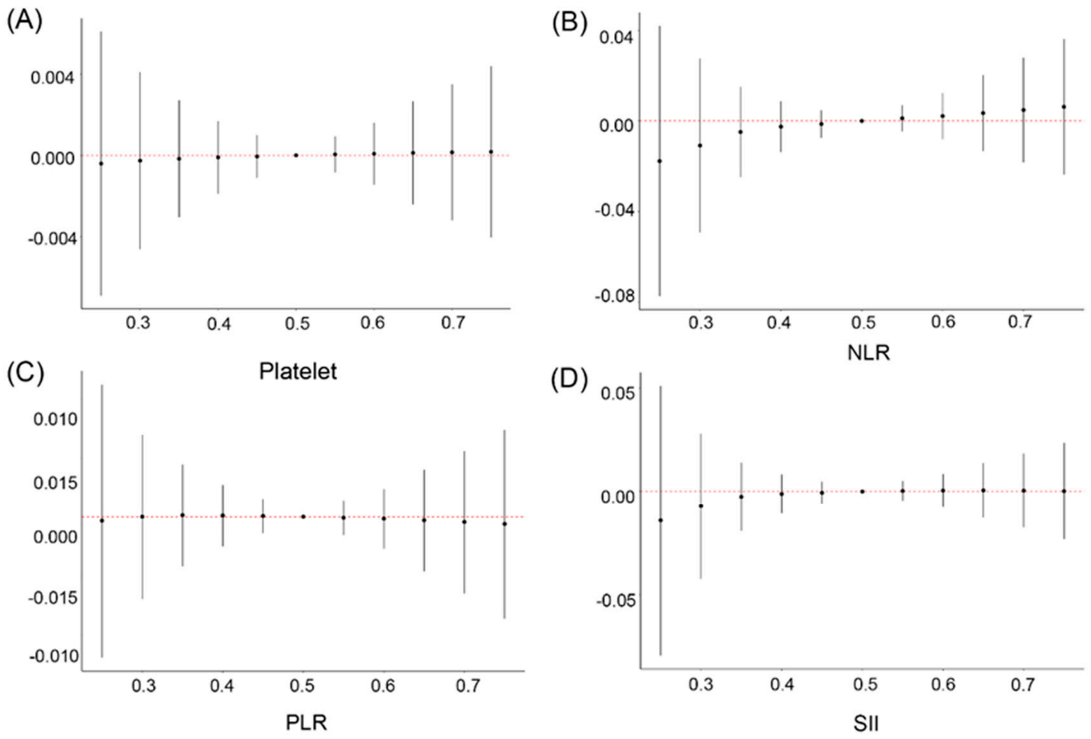

Fig S1. The overall association of screened OH-PAHs and PAEs mixtures with Platelet (A), NLR (B), PLR (C), and SII (D) was estimated by Bayesian kernel machine regression (BKMR). The model adjusted for maternal age, pre-pregnancy BMI, marital status, categorical education, race, smoking, passive smoking, exercise, and alcohol consumption

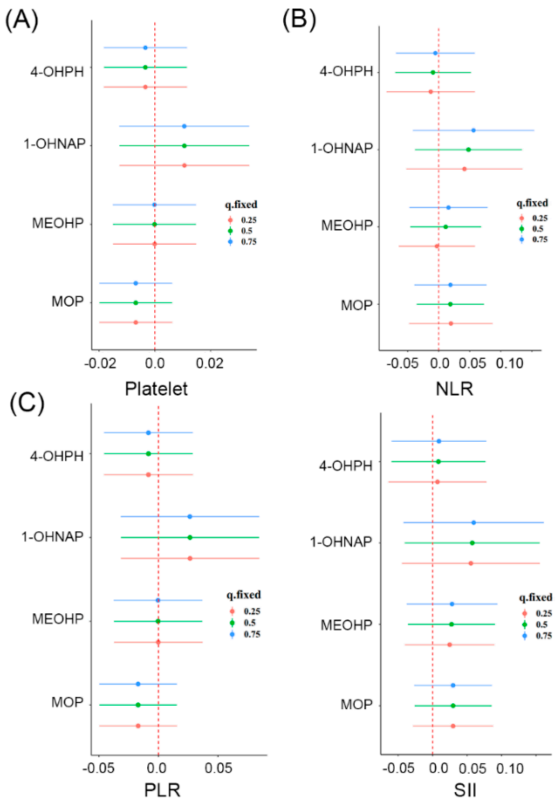

Fig S2. The single associations of screened OH-PAHs and PAEs with Platelet (A), NLR (B), PLR (C), and SII (D) were estimated by the

Bayesian kernel machine regression (BKMR) model. The model adjusted for maternal age, pre-pregnancy BMI, marital status, categorical education, race, smoking, passive smoking, exercise, and alcohol consumption.

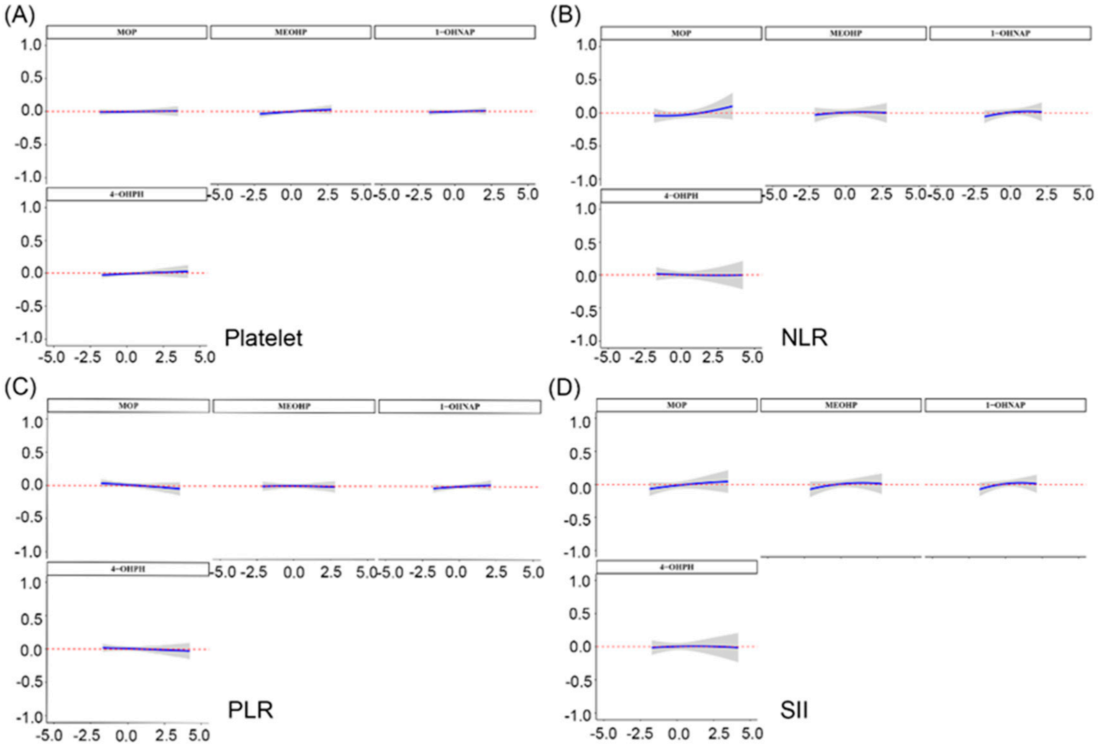

Fig S3. Univariate exposure-response relationships between the concentration of each substance and platelet (A), NLR (B), PLR (C), and SII (D) when the other substances were fixed at median concentrations. The model adjusted for maternal age, pre-pregnancy BMI, marital status, categorical education, race, smoking, passive smoking, exercise, and alcohol consumption.

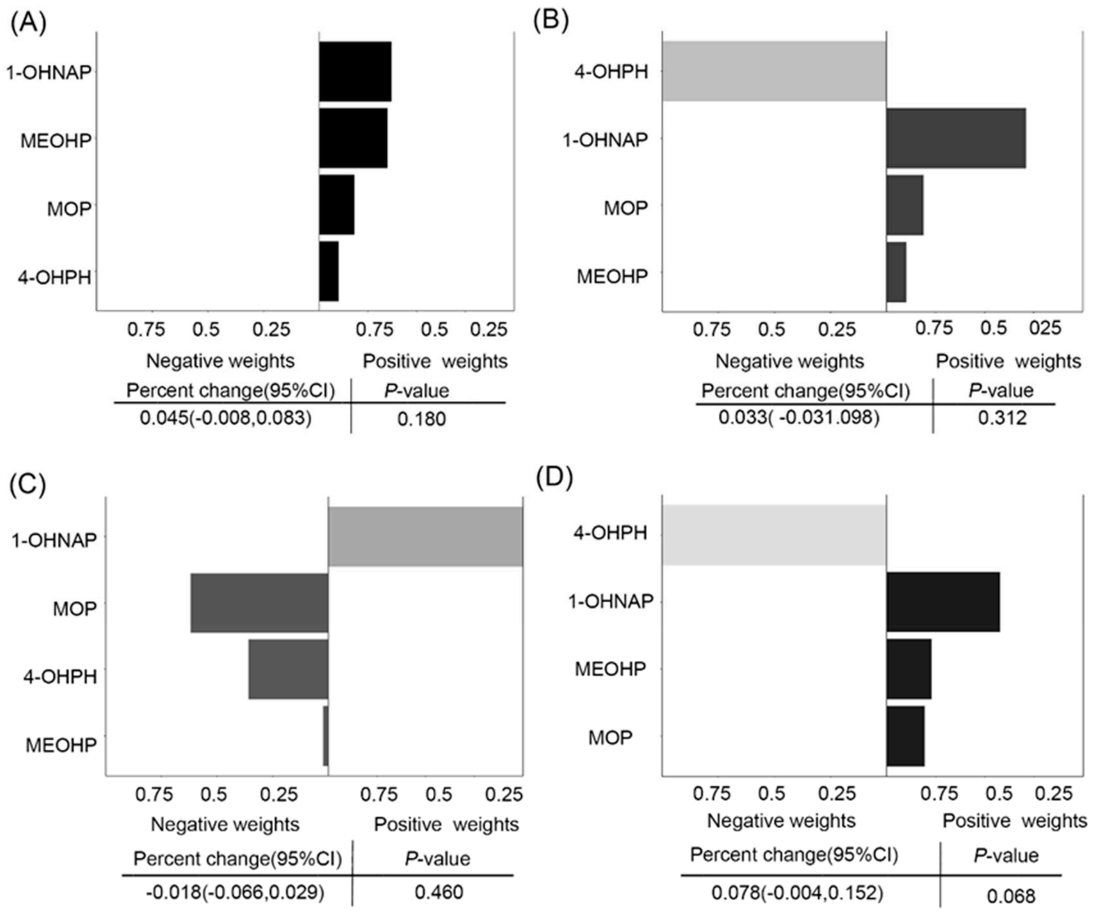

Fig S4 Estimation of the combined association of screening the four mixtures of OH-PAHs and PAE DNA s in blood platelet (A), NLR (B), PLR (C), and SII (D) and scaled weights corresponding to the proportion of the joint association for each chemical in Quantile g-computation.

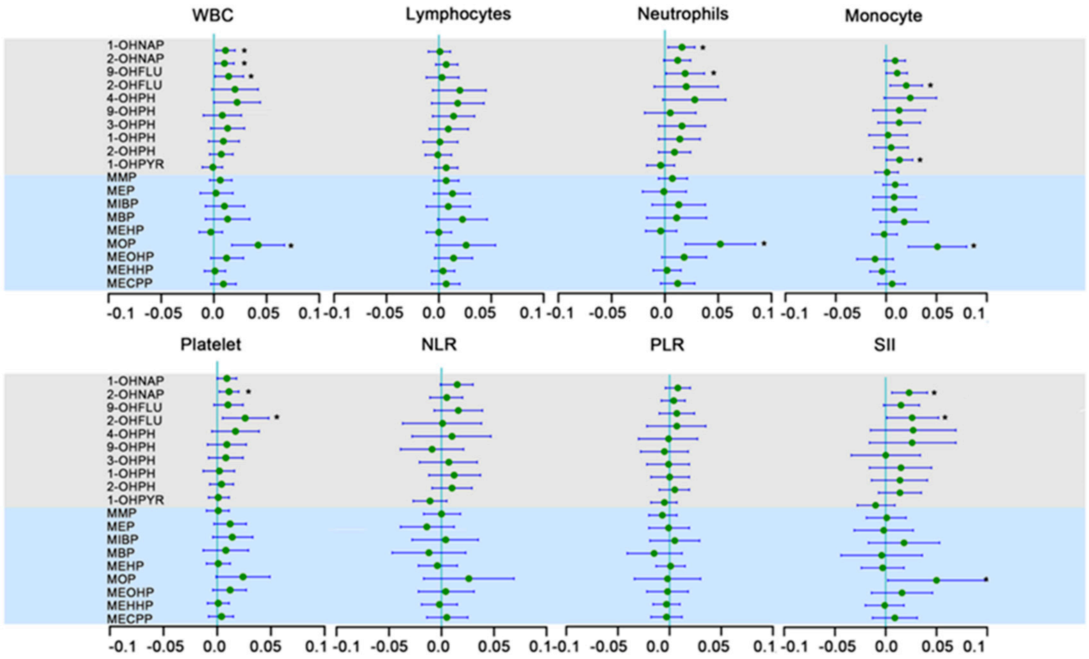

Fig S5. When we excluded the pregnant women with WBC < 4.0 × 109/L or > < 12.0 × 109/L,

the association of OH-PAHs or PAEs with blood cell-based biomarkers of inflammation in the urine of pregnant women. Linear regression models adjusted for maternal age, pre-pregnancy BMI, marital status, categorical education, race, smoking, passive smoking, exercise, and alcohol consumption.
